# The muscarinic antagonists scopolamine and atropine are competitive antagonists at 5-HT_3_ receptors

**DOI:** 10.1016/j.neuropharm.2016.04.027

**Published:** 2016-09

**Authors:** Martin Lochner, Andrew J. Thompson

**Affiliations:** a Department of Chemistry and Biochemistry, University of Bern, Freiestrasse 3, Bern, CH-3012, Switzerland; b Department of Pharmacology, Tennis Court Road, Cambridge, CB2 1PD, UK

**Keywords:** 5-HT_3_, Cys-loop, Binding site, Ligand docking, Scopolamine, Muscarinic, Antagonist, Muscarinic, Anxiety, Cognition, Memory, Depression, Hippocampus, Amygdala

## Abstract

Scopolamine is a high affinity muscarinic antagonist that is used for the prevention of post-operative nausea and vomiting. 5-HT_3_ receptor antagonists are used for the same purpose and are structurally related to scopolamine. To examine whether 5-HT_3_ receptors are affected by scopolamine we examined the effects of this drug on the electrophysiological and ligand binding properties of 5-HT_3_A receptors expressed in *Xenopus* oocytes and HEK293 cells, respectively. 5-HT_3_ receptor-responses were reversibly inhibited by scopolamine with an *IC*_50_ of 2.09 μM. Competitive antagonism was shown by Schild plot (p*A*_2_ = 5.02) and by competition with the 5-HT_3_ receptor antagonists [^3^H]granisetron (*K*_i_ = 6.76 μM) and G-FL (*K*_i_ = 4.90 μM). The related molecule, atropine, similarly inhibited 5-HT evoked responses in oocytes with an *IC*_50_ of 1.74 μM, and competed with G-FL with a *K*_i_ of 7.94 μM. The reverse experiment revealed that granisetron also competitively bound to muscarinic receptors (*K*_i_ = 6.5 μM). In behavioural studies scopolamine is used to block muscarinic receptors and induce a cognitive deficit, and centrally administered concentrations can exceed the *IC*_50_ values found here. It is therefore possible that 5-HT_3_ receptors are also inhibited. Studies that utilise higher concentrations of scopolamine should be mindful of these potential off-target effects.

## Highlights

• Muscarinic ligands scopolamine and atropine also have micromolar affinity at 5-HT_3_ receptors.
• The 5-HT_3_ receptor ligand granisetron also has micromolar affinity at muscarinic receptors.
• Scopolamine has a tetrahedral carbon linker that is responsible for its lower affinity at 5-HT_3_ receptors.
• Scopolamine is used as a preclinical model for inducing cognitive dysfunction.
• Use of high concentrations may inhibit 5-HT_3_ receptors and complicate analysis.

## Graphical abstract

**Figure:**
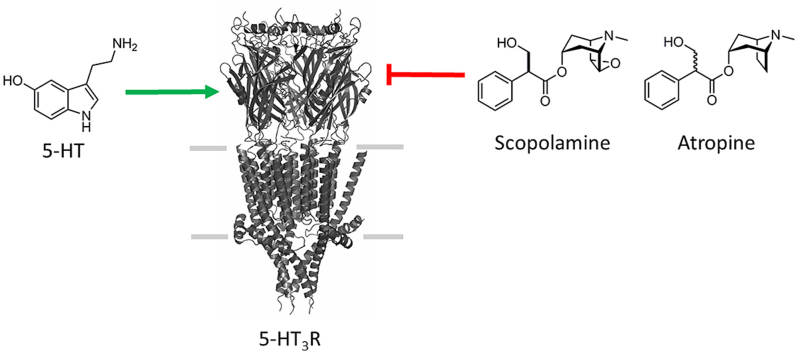

## 1 Introduction

Scopolamine is a high-affinity (nM) muscarinic antagonist that is used to treat post-operative nausea and vomiting, and motion sickness. As a research tool it is often administered to induce cognitive dysfunction. At higher doses it can also produce amnesia and compliance (Klinkenberg and Blokland, 2010). Atropine is a related muscarinic antagonist from the same biosynthetic pathway as scopolamine and is used as a cycloplegic and mydriatic in ophthalmology, and for the treatment of bradychardia.

Scopolamine readily passes the blood brain barrier and it is believed that inhibition of muscarinic receptors in the central nervous system causes a cholinergic deficit that impairs memory (Klinkenberg and Blokland, 2010). As an age-related deterioration in cognitive function is thought to be predominantly related to a decline in cholinergic neurotransmission, scopolamine administration has often been used to model dementia (Bartus, 2000). Scopolamine has therefore been extensively used for preclinical and clinical testing of treatments for cognitive impairment (Bartolomeo et al., 2000, Blin et al., 2009, Liem-Moolenaar et al., 2011).

In the clinic, 5-HT_3_ antagonists are mainly used for the treatment of nausea and vomiting following cancer therapy and general anaesthesia (Thompson, 2013, Walstab et al., 2010). Experimentally, they can also be administered to reverse scopolamine-evoked learning and memory deficits (Barnes et al., 1990, Chugh et al., 1991, Carli et al., 1997). In the brain 5-HT_3_ receptors are widely distributed in the amygdala and hippocampus, regions of critical importance in memory and spatial navigation, and involved in the control of emotional responses and their associated disorders such as anxiety and depression (Gulyas et al., 1999, Thompson and Lummis, 2007, Walstab et al., 2010). It is thought that the reversal of scopolamine-induced cognitive dysfunction by 5-HT_3_ receptor antagonists occurs by inhibiting pre-synaptic 5-HT_3_ receptors that modulate the functions of other neurotransmitters such as acetylcholine, dopamine, γ-aminobutyric acid and glutamate in this region (Seyedabadi et al., 2014). A similar mechanism is thought to underlie the anti-anxiolytic and anti-depressive actions of 5-HT_3_ antagonists.

**Fig. 1:**
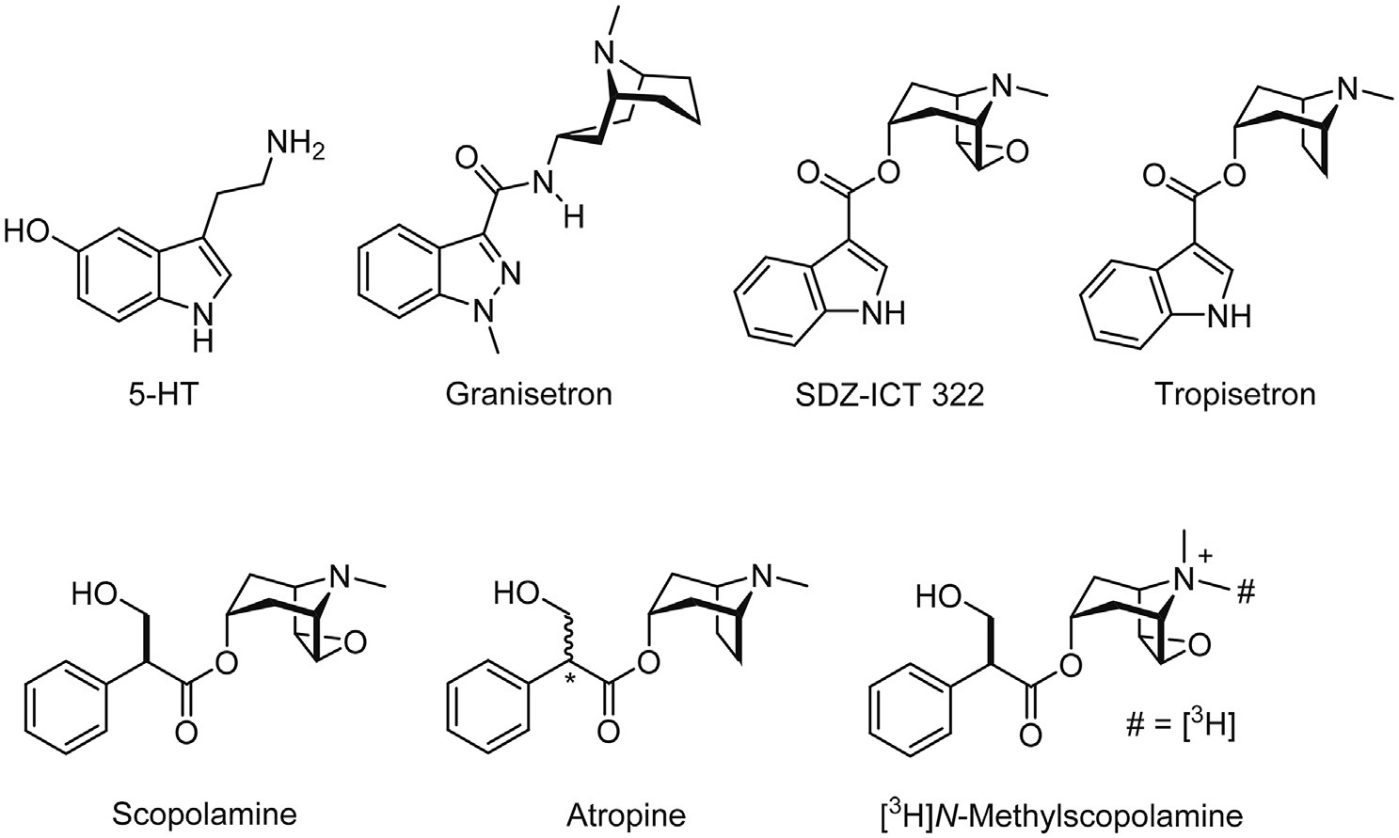
Chemical structures of endogenous agonist 5-HT, 5-HT_3_ receptor antagonists granisetron, tropisetron and SDZ-ICT 322, scopolamine, atropine and the radioligand [^3^H]*N*-methylscopolamine. Note that scopolamine is a single enantiomer whereas atropine is a mixture of epimers at the indicated (asterisk) carbon atom.

5-HT_3_ receptors are members of the Cys-loop family of ligand-gated ion channels (LGIC). These are responsible for fast excitatory and inhibitory neurotransmission in the central and peripheral nervous systems. The family includes nicotininc acetylcholine (nACh), γ-aminobutyric acid (GABA_A_) and glycine receptors, which are all cell-surface, transmembrane ion channels. They consist of five subunits that surround a central ion-conducting pore, and each subunit contains three distinct functional regions that are referred to as the extracellular, transmembrane and intracellular domains. The orthosteric binding site (that occupied by the endogenous agonist) is located between the extracellular domains of adjacent subunits, and is formed by the convergence of three amino acid loops from the principal subunit (loops A – C) and three β-sheets (loops D – F) from the complementary subunit (Thompson et al., 2008). Agonist binding results in the opening of a central ion-conducting pore that is located within the transmembrane domain (Peters et al., 2010, Hassaine et al., 2014). Ligands bind to both domains, but the orthosteric binding site is the main drug target. These 5-HT_3_ receptor competitive antagonists have high affinities (nM) and conform to a pharmacophore that consists of an aromatic group coupled to an azabicyclic ring via a carbonyl linker (Fig. 1). Both atropine and scopolamine also have these structural features, suggesting that these muscarinic antagonists could also bind at 5-HT_3_ receptors (Thompson, 2013).

Here we use a combination of electrophysiology, radioligand binding, flow cytometry and *in silico* ligand docking to provide evidence that, in addition to its block of muscarinic receptors, scopolamine is also a competitive antagonist of 5-HT_3_ receptors.

## 2 Materials and methods

### 2.1 Materials

Atropine and scopolamine were from Sigma-Aldrich (St. Louis, MO, USA). [^3^H]*N*-methylscopolamine (84 Ci/mmol) was from Perkin Elmer (Boston, MA, USA). Human 5-HT3A (Accession: 46,098) subunit cDNA was kindly provided by J. Peters (Dundee University, UK).

### 2.2 Oocyte maintenance

*Xenopus laevis* oocytes were purchased from EcoCyte Bioscience (Castrop-Rauxel, Germany) and maintained according to standard methods (Goldin, 1992) in ND96 (96 mM NaCl, 2 mM KCl, 1 mM MgCl_2_, 5 mM HEPES, pH 7.4).

### 2.3 Cell culture

Human embryonic kidney (HEK) 293 cells were grown on 90 mm round tissue culture plates as monolayers in DMEM/F12 (Gibco, Life Technologies, CA, USA) supplemented with 10% fetal bovine serum (FBS; Sigma Aldrich) at 37 °C in a moist atmosphere containing 5% CO_2_.

### 2.4 5-HT_3_ receptor expression

5-HT3A subunit cDNA was cloned into pGEMHE for oocyte expression. cRNA was *in vitro* transcribed from linearised plasmid cDNA template using the mMessage mMachine Ultra T7 Transcription kit (Ambion, Austin, Texas, USA). Stage V and VI oocytes were injected with 50 nl of 100–600 ng/μl cRNA (5–30 ng injected), and currents were recorded 1–3 days post-injection.

5-HT3A subunit cDNA was cloned into pcDNA3.1 for expression in HEK 293 cells. Cells were transiently transfected with this cDNA using polyethyleneimine (PEI: 25 kDa, linear, powder, Polysciences Inc., Eppelheim, Germany). 30 μl of PEI (1 mg ml^−1^), 5 μg cDNA and 1 ml DMEM were incubated for 10 min at room temperature, added drop wise to a 90 mm plate, at 80–90% confluency, and incubated for 2–3 days before harvesting.

### 2.5 Muscarinic receptor preparation

Muscarinic receptors were isolated from the cerebral cortices of adult male Guinea pigs (200–300 g). Brains were dissected into 10 mM Tris-HCl + 1 mM EDTA (pH 7.6) on ice and homogenised using a Teflon-glass homogeniser with a motor-driven pestle (30 s, 300 rpm). The tissue was pelleted 17,000 g for 30 min and the membranes resuspended, and then centrifuged again using the same procedure. The final pellet was homogenised in 10 mM HEPES buffer (pH 7.4) and used directly for radioligand binding. Experiments involving animals were approved by the University of Cambridge Animal Welfare and Ethical Review Body (PHARM 004/15).

### 2.6 Radioligand binding

Saturation binding (8 point) curves were measured by incubating either crude extracts of HEK 293 cells stably expressing 5-HT_3_ receptors, or Guinea pig membrane preparations, in 0.5 ml incubations containing 10 mM HEPES buffer (pH 7.4) and 0.1–1 nM [^3^H]granisetron or 1–10 nM [^3^H]*N*-methylscopolamine. Competition binding (10 point) was determined by incubating the same receptors preparations in 0.5 ml HEPES buffer containing either 0.6 nM [^3^H]granisetron or 0.6 nM [^3^H]*N*-methylscopolamine, and differing concentrations of competing ligands. Non-specific binding was determined with 1 mM quipazine or 10 μM scopolamine respectively. Incubations were terminated by filtration onto Whatman GF/B filters (Sigma Aldrich) wetted with HEPES buffer + 0.3% polyethyleneimine, followed by two rapid washes with ice-cold HEPES buffer. Protein concentration was calculated using a Lowry protein assay with bovine serum albumin standards (Lowry et al., 1951). Radioactivity was measured using a Tri-Carb 2100 TR (Perkin Elmer, Waltham, MA, USA) scintillation counter.

### 2.7 Flow cytometry

HEK 293 cells expressing the 5-HT_3_ receptor were grown in monolayers and harvested from 90 mm culture dishes using 10 ml Trypsin-EDTA (Sigma Aldrich) for 10 min at 37 °C. Digestion was terminated by the addition of 25 ml DMEM +10% FBS and cells pelleted at low speed for 2 min. The pellet was resuspended in 3 ml phosphate buffered saline (PBS: 137 mM NaCl, 8.0 mM Na_2_HPO_4_, 2.7 mM KCl, 1.47 mM KH_2_PO_4_, pH 7.4) and cells filtered through a cell strainer (BD Falcon, Franklin Lakes, NJ, USA). Competition binding was measured by incubating HEK 293 cells with different concentrations of non-labeled ligands and 10 nM G-FL (Jack et al., 2015, Lochner and Thompson 2015). After 10 min incubation, cells were pelleted and rapidly washed in PBS before being resuspended in the same buffer and analysed on a BD Accuri C6 flow cytometer (Becton, Dickinson and Company, NJ, USA) at 488 nm excitation/530 nm emission.

### 2.8 Electrophysiology

Using two electrode voltage clamp, *Xenopus* oocytes were routinely clamped at −60 mV using an OC-725 amplifier (Warner Instruments, Connecticut, USA), NI USB-6341 X Series DAQ Device (National Instruments, Berkshire, UK) and the Strathclyde Electrophysiology Software Package (University of Strathclyde, UK). Micro-electrodes were fabricated from borosilicate glass (GC120TF-10, Harvard Apparatus, Edenbridge, Kent, UK) using a two stage horizontal pull (P-1000, Sutter Instrument Company, California, USA) and filled with 3 M KCl. Pipette resistances ranged from 0.7 to 1.5 MΩ. Oocytes were routinely perfused with ND96 at a rate of 15 ml min^−1^. Drug application was via a simple gravity fed system calibrated to run at the same rate. Antagonists were routinely co-applied in the presence of 2 μM 5-HT or continuously applied for 1 min before the co-application of 2 μM 5-HT. A 2 min wash was used between applications.

### 2.9 Data analysis

All data analysis was performed with GraphPad Prism v5.00 (GraphPad Software, San Diego, CA, USA). For concentration-response curves, peak currents were measured for each concentration of agonist and normalised to the maximal peak current in the same oocyte. For inhibition curves, the peak current response to 2 μM 5-HT was measured at in the absence or presence of antagonist and normalised to the response to 2 μM 5-HT alone. The mean and S.E.M. for a series of oocytes was plotted against agonist or antagonist concentration and iteratively fitted to the following equation:

(1) $$y=I_{\text{min}}+\frac{I_{\text{max}}-I_{\text{min}}}{1+10^{\log\left(EC_{50}-x\right)n_{H}}}$$

where *I*_min_ is the baseline current, *I*_max_ is the peak current evoked by agonist, *EC*_50_ is the concentration of agonist needed to evoke a half-maximal response, *x* is the ligand concentration and *n*_*H*_ is the Hill slope. *K*_b_ was estimated from *IC*_50_ values using the Cheng-Prusoff equation with the modification by Leff and Dougall (1993):

(2) $$K_{\text{b}}=\frac{IC_{50}}{\left({\left(2+{\left(\left[\text{A}\right]/\left[{\text{A}}_{50}\right]\right)}^{nH}\right)}^{1/nH}\right)-1}$$

where *K*_b_ is the dissociation constant of the competing drug, *IC*_50_ is the concentration of antagonist required to half the maximal response, [A] is the agonist concentration, [A_50_] is the agonist *EC*_50_, and *n*_*H*_ is the Hill slope of the agonist.

Analysis of competitive inhibition was performed by Schild Plot according to the following equation:

(3) $$\text{log}\left[\left({\text{EC}}_{50}^{\prime }/{\text{EC}}_{50}\right)-1\right]\;=\;\text{log}\left[\text{L}\right]-{\text{logK}}_{\text{b}}$$

where ${\text{EC}}_{50}^{\prime }$ and *EC*_50_ are values in the presence and absence of antagonist (Dose Ratio), [*L*] is the concentration of antagonist, and *K*_b_ is the equilibrium dissociation constant for the antagonist receptor interaction. Further analysis was performed using the Gaddum-Schild equation (slope = 1) as recommended by Neubig et al. (2003):

(4) $$\text{p}EC_{50}=-\log\left(\left[L\right]+10^{-pA_{2}}\right)-\log\;C$$

where p*EC*_50_ is the negative logarithm of the agonist *EC*_50_, [*L*] is the antagonist concentration, log*C* is a constant and p*A*_2_ is the negative logarithm of the antagonist concentration needed to double the concentration of agonist required in order to elicit a response that is comparable to the original response in the absence of antagonist. p*A*_2_ is equal to the negative logarithm of *K*_b_ when the slope of the Schild plot is exactly 1.

Kinetic parameters were determined according to the following model of a simple bimolecular binding scheme:

(5) $$L+R\overset{k_{on}}{\underset{k_{off}}{⇌}}LR$$

where *L* is the free ligand concentration, *R* is receptor concentration, *LR* is the ligand-receptor complex and *k*_on_ and *k*_off_ are the microscopic association and dissociation rate constants. In a simple scheme such as this, the equilibrium dissociation constant (*K*_d_) is equal to the ratio of dissociation to association rate constants, such that:

(6) $$K_{\text{d}}=\frac{k_{off}}{k_{on}}$$

According to a one site binding model of the type shown, the time constants for the onset and recovery of an antagonist response can be used to estimate *k*_+1_ and *k*_-1_:

(7) $$\frac{1}{\tau_{\text{off}}}{=\;\text{k}}_{-1}$$

and

(8) $$\frac{1}{\tau_{\text{on}}}{=\;\text{k}}_{+1}{\left[\text{L}\right]+\text{k}}_{-1}$$

where τ_on_ refers to the time constant for the onset of inhibition, τ_off_ refers to recovery from inhibition and [*L*] is antagonist concentration.

Competition binding data were analysed by iterative curve fitting according to:

(9) $$y=A_{\min}\frac{A_{\max}-A_{\min}}{1+10^{\left[L\right]-\log\;IC_{50}}}$$

*K*_i_ values were determined from the *IC*_50_ values using the Cheng-Prusoff equation:

(10) $$K_{\text{i}}=\frac{IC_{50}}{1+\left[L\right]/K_{d}}$$

where *K*_i_ is the equilibrium dissociation constant for binding of the unlabeled ligand, [*L*] is the concentration of labeled ligand and *K*_d_ is the equilibrium dissociation constant of the labeled ligand.

### 2.10 Docking

We used a template of granisetron bound to 5HTBP (PDB ID 2YME); an AChBP chimaera with substitutions in the binding site that mimic the 5-HT_3_ receptor (Kesters et al., 2013). The three-dimensional structure of the hydrochloride salt of scopolamine was extracted from the Cambridge Structural Database (CSD, access code KEYSOW) and Chem3D Pro v14.0 (CambridgeSoft, Cambridge, UK) was used to construct scopolamine based on the crystal structure. The generated ligand was subsequently energy-minimised using the implemented MM2 force field. Similarly, construction of the three-dimensional structure of the protonated form of tropisetron was based on the crystal structure of *N*-methyl tropisetron (CSD access code BEGLEG), and the three-dimensional structure of SDZ-ICT 322 was based on the crystal structures of *N*-methyl tropisetron (for the indole carboxylic moiety; CSD access code BEGLEG) and scopolamine (for the tricyclic scopine moiety; CSD access code KEYSOW), followed by energy-minimisation. The binding site was defined as being within 10 Å of the centroid of the aromatic side-chain of W183, a residue that is centrally located in the binding site and is important for the binding of other 5-HT_3_ competitive ligands. The ligands were docked into this site using GOLD Suite v5.3 (The Cambridge Crystallographic Data Centre, Cambridge, UK) with the GoldScore function and default settings. For docking, scopolamine was defined as flexible, while the C—C bond between the ester group and the aromatic indole ring of SDZ-ICT322 and tropisetron was defined as rigid due to conjugation. Ten docked poses were generated for each ligand and the poses visualized with PyMol v1.7.5.0.

## 3 Results

### 3.1 Effects of scopolamine on 5-HT_3_ receptor currents

**Fig. 2:**
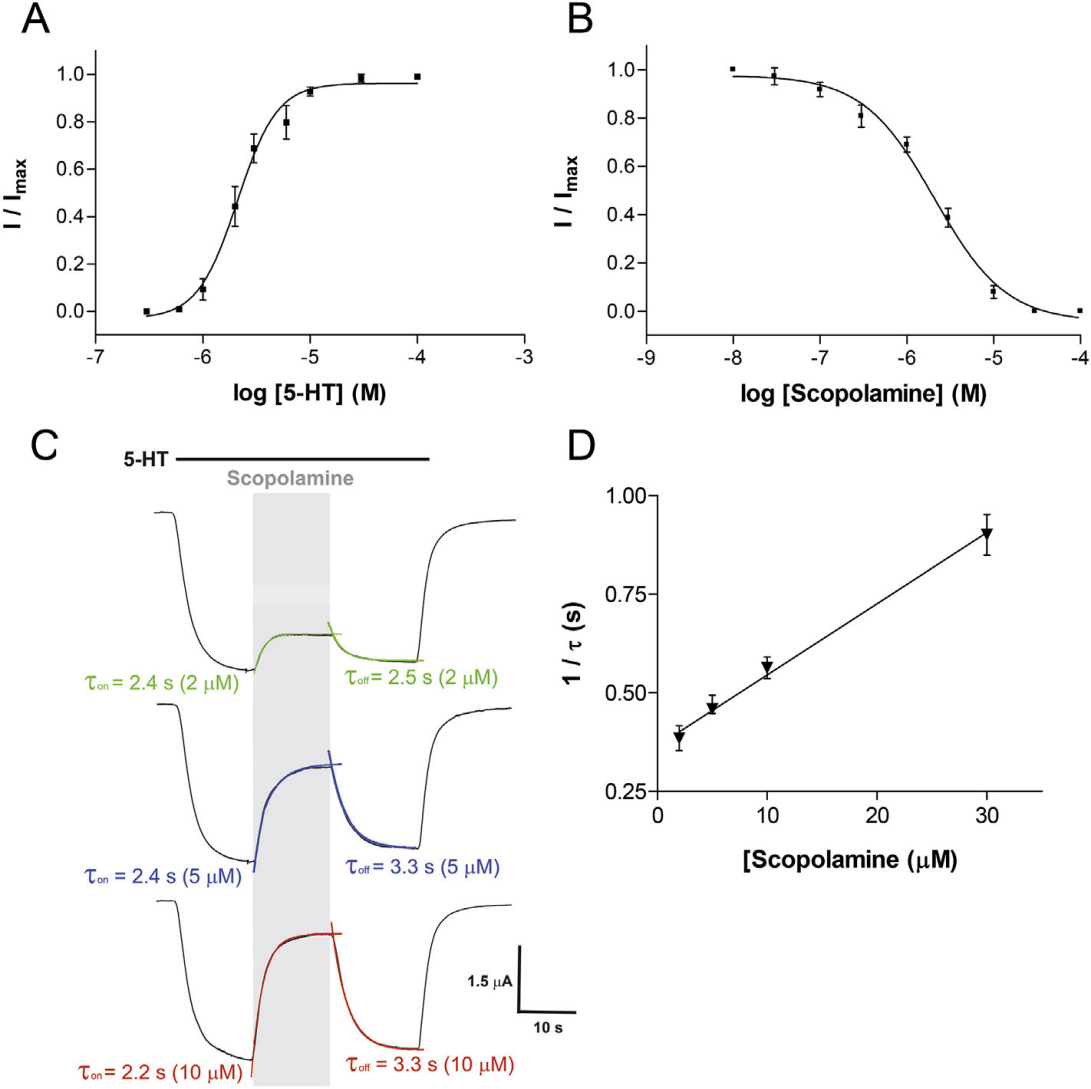
The effect of scopolamine on 5-HT_3_ receptor currents. (**A**) Concentration-response curve for 5-HT. (**B**) Concentration-inhibition of the 2 μM 5-HT response by co-applied scopolamine. The data in 2A are normalised to the maximal peak current response for each oocyte and represented as the mean ± S.E.M. for a series of oocytes. In Fig. 2B, inhibition by scopolamine is shown relative to the peak current response to 2 μM 5-HT alone. For 5-HT curve fitting yielded a p*EC*_50_ of 5.65 ± 0.02 (*EC*_50_ = 2.24 μM, *n* = 6) and Hill slope of 2.06 ± 0.14. The p*IC*_50_ value for scopolamine was 5.68 ± 0.05 (*IC*_50_ = 2.09 μM, *n* = 6) with a Hill Slope of 1.06 ± 0.05. (**C**) Sample traces showing the onset (τ_on_) and recovery (τ_off_) of scopolamine inhibition (*grey bar*) during a 2 μM 5-HT application (*filled bar*). (**D**) Onset of inhibition was well fitted by mono-exponential functions to give *k*_obs_ (*n* = 17). A plot of the reciprocal of these time constants versus the scopolamine concentration showed a linear relationship where the slope = *k*_on_ (2.60 × 10^4^ M^−1^ s^−1^) and the *y*-axis intercept = *k*_off_ (0.32 s^−1^).

Application of 5-HT to *Xenopus* oocytes expressing the 5-HT_3_ receptor produced concentration-dependent, rapidly activating, inward currents that slowly desensitised (τ = 42 ± 5 s; *n* = 8) over the time-course of the applications. Plotting peak current amplitude against a series of 5-HT concentrations allowed the data to be fitted with Eq. (1) to give a p*EC*_50_ of 5.65 ± 0.02 (*EC*_50_ = 2.24 μM, *n* = 6) and Hill slope of 2.06 ± 0.14 (Fig. 2A). Agonist responses were completely inhibited by the established 5-HT_3_ receptor-specific antagonist granisetron (100 nM, *data not shown*). Uninjected oocytes did not respond to 5-HT.

Application of scopolamine to oocytes expressing 5-HT_3_ receptors did not elicit a response when applied alone, but caused a concentration-dependent inhibition of the response during a co-application of 2 μM 5-HT (Fig. 2). The p*IC*_50_ value for scopolamine was 5.68 ± 0.05 (*IC*_50_ = 2.09 μM, *n* = 6) with a Hill Slope of 1.06 ± 0.05. This gave a *K*_b_ of 3.23 μM (Eq. (2)). The same concentration-dependent effect was also seen when scopolamine was applied during the 5-HT application (Fig. 2C). Using this protocol the onset of inhibition could be fitted with a mono-exponential function and the reciprocal plotted against antagonist concentration to yield association (slope; *k*_on_ = 2.60 × 10^4^ M^−1^ s^−1^) and dissociation (*y*-axis intercept; 0.32 s^−1^) rates that gave a *K*_d_ of 12.3 μM (Fig. 2D, Eq. (6)). Inhibition was fully reversible after 1 min of washing and was unaltered by a 1 min scopolamine pre-application (*data not shown*).

### 3.2 Mechanism of scopolamine block

**Fig. 3:**
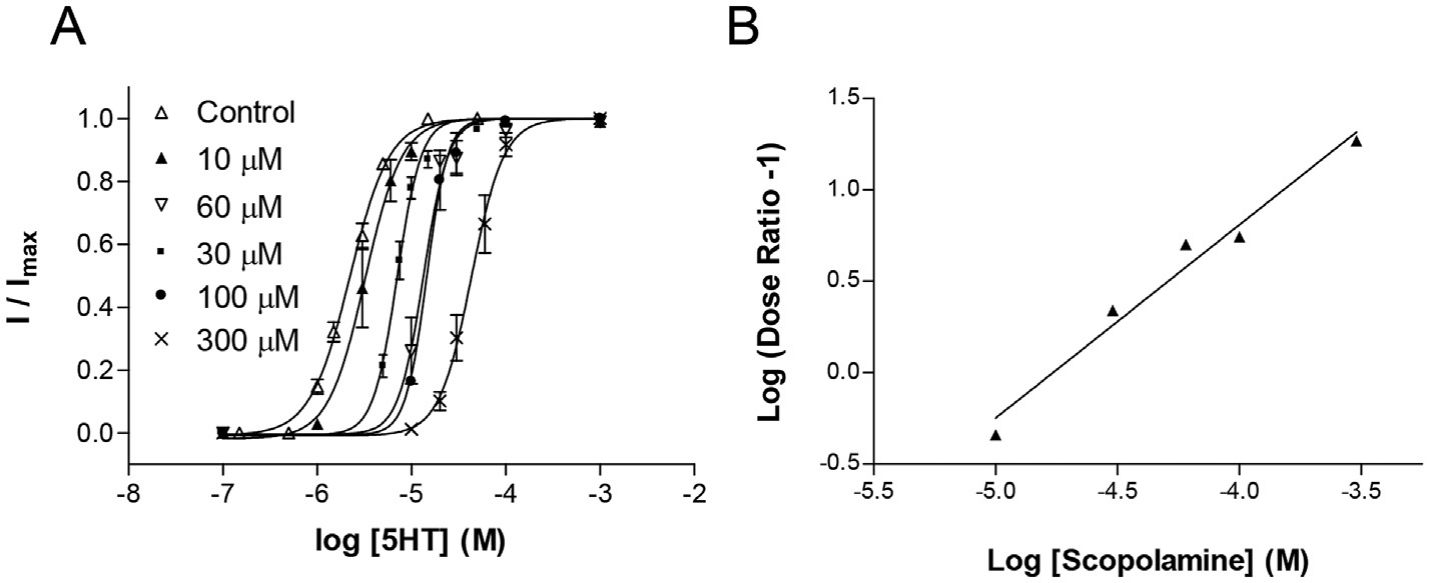
The mechanism of 5-HT_3_ receptor inhibition by scopolamine. (**A**) Concentration-response curves were performed in the absence or presence of the indicated concentrations of scopolamine. The curves showed parallel dextral shifts with maximal currents restored by increasing concentrations of 5-HT. Parameters derived from these curves can be seen in Table 1. (**B**) A Schild plot was created from the dose ratios of the curves shown in 3A and fitted with Eq. (3) to yield a slope of 1.06 ± 0.10 (R^2^ = 0.97) and a p*A*_2_ of 5.03 ± 0.43 (*K*_b_, 9.33 μM).

Increasing concentrations of scopolamine (10 μM, 30 μM, 60 μM, 100 μM, 300 μM) caused a parallel rightward shift in the 5-HT concentration-response curve, with no change in the maximal response (Fig. 3A, Table 1). A Schild plot of these results (Fig. 3B), yielded a gradient close to 1 (1.06 ± 0.10, R^2^ = 0.97) and a p*A*_2_ value of 5.03 ± 0.43 (*K*_b_ = 9.33 μM). The *K*_b_ estimate was similar (2.88 μM) if the data were fitted using a nonlinear regression method (Eq. (4)) as recommended by Neubig et al. (2003) and Lew and Angus (1995). These data support a competitive mechanism of action, indicating that scopolamine binds to the orthosteric binding site.

### 3.3 Binding at 5-HT_3_ and muscarinic receptors

**Fig. 4:**
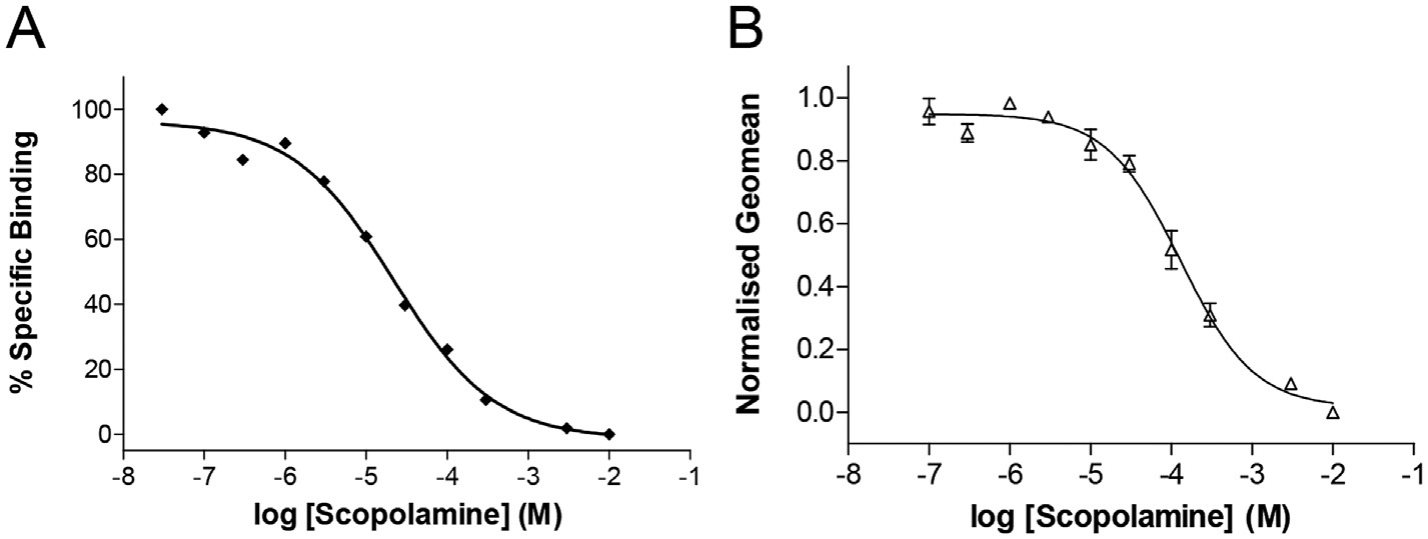
Competition of scopolamine with an established 5-HT_3_ receptor antagonist. (**A**) Radioligand binding curves for the competition of 0.6 nM [^3^H]granisetron and varying concentrations of scopolamine at crude membrane extracts of 5-HT_3_ receptors from stably expressing HEK 293 cells. Data was normalised to [^3^H]granisetron binding in the absence of antagonist and fitted with Eq. (10). The curve is representative of 3 similar experiments, which gave an average p*K*_i_ of 5.17 ± 0.24 (*K*_i_ = 6.76 μM, *n* = 3). (**B**) Flow cytometry, showing the competition of 10 nM G-FL (a fluorescent derivative of granisetron; Jack et al., 2015) and varying concentrations of scopolamine at 5-HT_3_ receptors expressed on the surface of live HEK 293 cells. The average p*K*_i_ of these experiments was similar to values from radioligand competition (5.31 ± 0.09, *K*_i_ = 4.90 μM, *n* = 8).

To further test for a competitive binding at the 5-HT_3_ receptor, we measured competition of unlabelled scopolamine with [^3^H]granisetron, an established high-affinity competitive antagonist at these receptors. Scopolamine displayed concentration-dependent competition with 0.6 nM [^3^H]granisetron (∼*K*_d_, Fig. 4), yielding an average p*K*_i_ (Eq. (10)) of 5.17 ± 0.24 (Fig. 4; *K*_i_ = 6.76 μM, *n* = 3).

Saturation binding using radiolabelled scopolamine was also undertaken at 5-HT_3_ receptors. Although the *K*_i_ of scopolamine was too low to accurately measure binding, the compound [^3^H]*N*-methylscopolamine that we used contains a permanent quaternary amine that increases its affinity at nicotinic receptors (Fig. 1, Schmeller et al., 1995). However, at concentrations of up to 10 nM, no saturable binding was observed for this radioligand at 5-HT_3_ receptors.

Competition of scopolamine was also measured at 5-HT_3_ receptor by flow cytometry with a fluorescently labeled form of granisetron (G-FL (Jack et al., 2015),). Concentration-dependent competition of G-FL with scopolamine gave an average p*K*_i_ (Eq. 11) of 5.31 ± 0.09 (Fig. 4; *K*_i_ = 4.90 μM, *n* = 8). This is similar to the affinities measured using electrophysiology and radioligand binding and provides further support for a competitive mode of action.

In the reverse experiment, competition binding of granisetron with [^3^H]*N*-methylscopolamine was examined at muscarinic receptors. The *IC*_50_ for granisetron at muscarinic receptors was 14.1 ± 3.1 μM (*n* = 7), yielding a *K*_i_ of 6.5 μM (Eq. (10)).

### 3.4 Properties of atropine

**Fig. 5:**
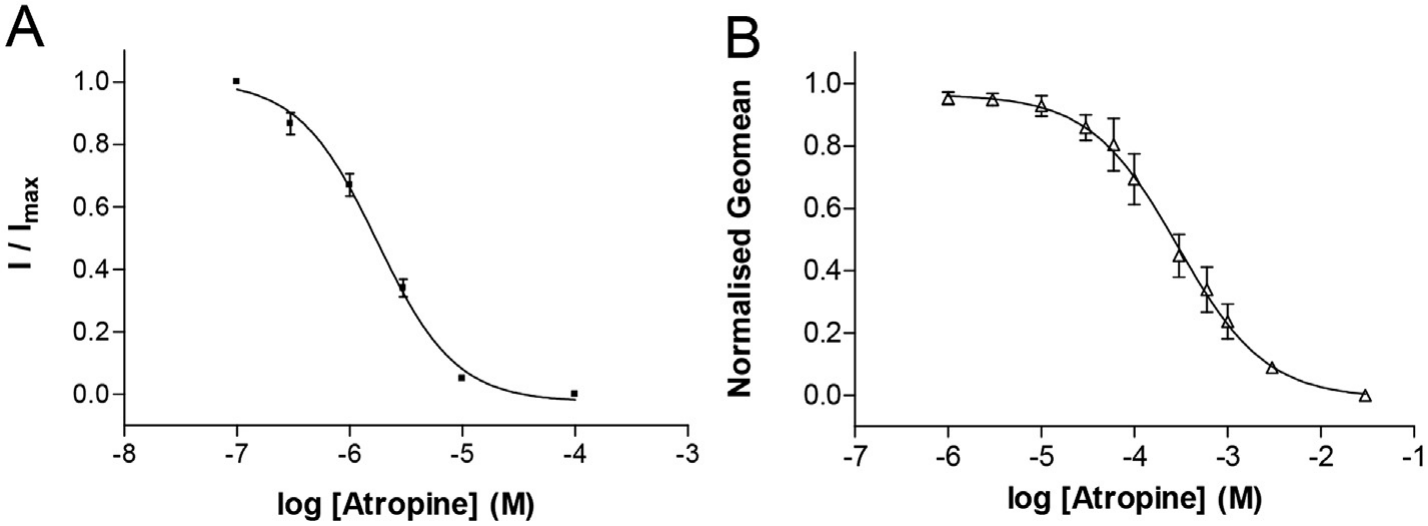
Effects of atropine on the electrophysiological responses to 5-HT and binding of G-FL. (**A**) Concentration-inhibition of the 2 μM 5-HT response by co-applied atropine. For each oocyte the responses in the presence of antagonist are normalised to the peak current response to 5-HT alone and data represented as the mean ± S.E.M. for a series of oocytes. Curve fitting yielded a p*IC*_50_ of 5.76 ± 0.14 (*IC*_50_ = 1.74 μM, *n* = 5) and Hill Slope of 1.06 ± 0.05. (**B**) Flow cytometry, showing the competition of 10 nM G-FL (a fluorescent derivative of granisetron; Jack et al., 2015) and varying concentrations of atropine at 5-HT_3_ receptors expressed on the surface of live HEK 293 cells. The affinity (p*K*_i_ = 5.10 ± 0.16, *K*_i_ = 7.94 μM, *n* = 5) of atropine calculated from these experiments was similar to that measured using electrophysiology.

Atropine is a structurally related muscarinic antagonist (Fig. 1). To test its pharmacological properties we performed measurements using electrophysiology and flow cytometry. In oocytes expressing 5-HT_3_ receptors, atropine did not elicit a response when applied alone, but it caused concentration-dependent inhibition of the 2 μM 5-HT-evoked response with a p*IC*_50_ of 5.76 ± 0.14 (*IC*_50_ = 1.74 μM, *n* = 5) and Hill Slope of 1.06 ± 0.05 (Fig. 5A). This yielded a *K*_b_ of 1.89 μM (Eq. (2)). Inhibition was fully reversible after 1 min of washing and was unaltered by pre-application (*data not shown*).

Competition of G-FL and atropine was also shown by flow cytometry (Fig. 5B). Concentration-dependent measurements were fitted to give a p*K*_i_ (Eq. (10)) of 5.10 ± 0.16 (*K*_i_ = 7.94 μM, *n* = 5).

### 3.5 Docking studies

**Fig. 6:**
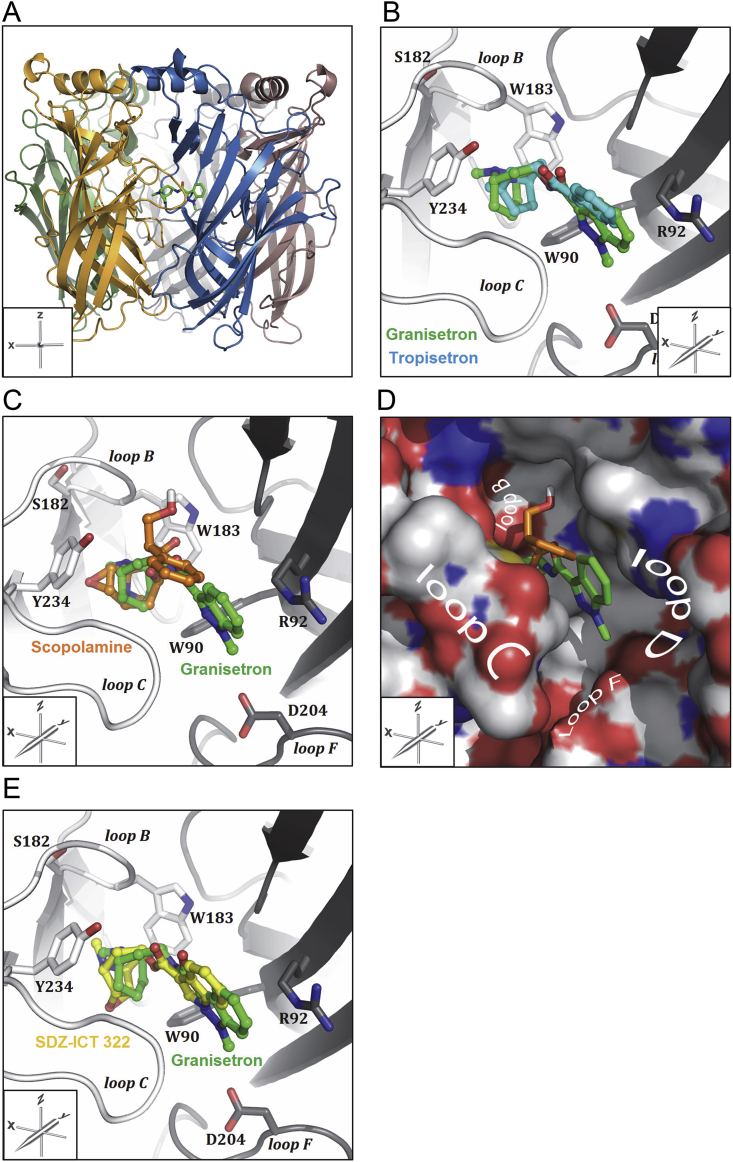
Representative examples of 5-HT_3_ receptor antagonists (ball-and-stick representation) docked into a 5-HT_3_ receptor orthosteric binding site model (PDB ID: 2YME; a co-crystal of granisetron bound to a mutant AChBP that contains residues from the 5-HT_3_ receptor binding site (termed 5HTBP; Kesters et al., 2013) and important binding site residues (stick representation). Principle face (left-hand side, light grey), complementary face (right-hand side, dark grey). (**A**) 2YME from the side (*y*-axis) showing the location of granisetron (green) in the orthosteric binding site at the interface of two adjacent subunits. (**B**) Proposed binding pose for tropisetron (blue) overlaying granisetron (green) from the co-crystal structure 2YME. (**C**) The proposed binding pose for scopolamine (orange) showing its orientation in the 5-HT_3_ binding site. (**D**) A surface representation of 5HTBP bound with granisetron and an overlay of docked scopolamine showing the hydroxyl of the carbonyl linker that, owing to steric constraints, is located within a cavity at the rear of the binding site. It can be seen that while the scopine head of scopolamine (orange) is at the same location as the azabicyclic rings of granisetron (green), the steric bulk, flexibility and presence of a hydroxyl in the linker region results in the aromatic ring being orientated away from loops D and F. (**D**) In contrast, the proposed binding pose for SDZ-ICT 322 (yellow) is more similar to that of granisetron. For chemical structures of the described ligands see Fig. 1.

Based upon the evidence that scopolamine binds at the orthosteric binding site we used a bio-informatics approach to probe possible ligand orientations and try to understand why the affinity of scopolamine was lower than other established 5-HT_3_ receptor antagonists. To this end we chose a crystal structure of a 5-HT_3_ receptor-AChBP chimera (termed 5HTBP) complexed with granisetron (PDB ID: 2YME) as a binding site model (Fig. 6A, Kesters et al., 2013). For the purpose of validation we first removed granisetron from the template and re-docked both this ligand and the closely related 5-HT_3_ receptor antagonist, tropisetron, into the binding site template. The proposed ligand orientations of these two antagonists were almost identical to the binding pose from the crystal structure 2YME. This is illustrated in Fig. 6B where tropisetron is shown with its bicyclic moiety located between the aromatic side chains of W90, W183 and Y234 and the flat indole ring is sandwiched between loop C and R92 from loop D.

Following from our docking with established 5-HT_3_ antagonists, we performed docking with scopolamine. This yielded a docked pose cluster (Fig. 6C) that placed the scopine head of scopolamine at the same location as the azabicyclic rings of granisetron and tropisetron, but owing to the flexibility of scopolamine and the steric restraints imposed by the tight binding cavity, the hydroxyl of the carbonyl linker was extended into a pocket at the rear of the binding site, displacing the aromatic ring by ∼3 Å towards the principal binding interface (Fig. 6D).

SDZ-ICT 322 (Fig. 1), is a competitive, highly potent 5-HT_3_ receptor antagonist that contains key structural elements of both scopolamine and high affinity 5-HT_3_ receptor antagonists such as granisetron and tropisetron (Blum et al., 1992); it has the same tricyclic scopine moiety as scopolamine, which is rigidly linked to the flat heteroaromatic group (indole) found in granisetron and tropisetron. Docking of SDZ-ICT 322 into the 5-HT_3_ receptor binding site predicted an orientation similar to granisetron and tropisetron, with its aromatic indole group close to the side chain of R92 from loop D and the scopine tricycle pointing towards the β-sheets of the principal face, surrounded by the aromatic rings of W90, W183 and Y234 (Fig. 6E).

## 4 Discussion

This study describes the effects of scopolamine and atropine on human 5-HT_3_ receptors. Both compounds were antagonists with μM potencies. For scopolamine, binding at the orthosteric site was demonstrated by Schild analysis and competition with the 5-HT_3_ receptor antagonists [^3^H]granisetron and G-FL. *In silico* docking predicted that molecular features of the carbonyl linker of scopolamine may alter its orientation within the binding site and could account for the lower potency when compared to established 5-HT_3_ receptor antagonists. Evidence for this is discussed in more detail below.

The observation that scopolamine competitively inhibits 5-HT_3_ receptor responses was anticipated as it has structural similarities with other 5-HT_3_ receptor antagonists (Fig. 1) and ligand promiscuity at 5-HT_3_ receptors has been reported elsewhere. For example, epibatidine and tropisetron are high affinity agonists of α7 nACh and high affinity antagonists of 5-HT_3_ receptors. Similarly, 5-HT_3_ receptors also have lower affinity competitive interactions with dopamine, acetylcholine, nicotine, *d*-tubocurarine, chloroquine, varenecline and strychnine, as well as allosteric modulators such as anaesthetics, alcohols, steroids and terpenoids and the non-competitive antagonists picrotoxin, ginkgolides and mefloquine (Thompson and Lummis, 2008, Thompson and Lummis, 2013, Thompson et al., 2014). It is perhaps more surprising that the affinities of scopolamine and atropine were not higher given their structural similarities to 5-HT_3_ receptor antagonists that bind with nM affinities. However, the lower affinities are likely to result from both scopolamine and atropine having an aromatic ring that is not directly attached to the ester moiety that forms the link with the bicyclic amine, a bond that is common to all 5-HT_3_ receptor antagonists (Thompson, 2013). The direct conjugation of the carbonyl (ester or amide) group with the aromatic ring provides 5-HT_3_ receptor antagonists with planarity and rigidity that is crucial for potent inhibition and high-affinity binding (Hibert, 1994). Instead, scopolamine and atropine have linkers that contain a tetrahedral carbon that carries a polar hydroxymethyl substituent (Fig. 1). The importance of this region is highlighted by SDZ-ICT 322, a ligand that is also a high affinity 5-HT_3_ receptor antagonist (p*A*_2_ = 10.6 in isolated rabbit vagus nerve, p*K*_d_ = 9.2 in N1E cells) but has the same scopine tricyclic moiety as scopolamine directly linked to the aromatic indole ring (Blum et al., 1992). This hypothesis is further supported by the low affinity of atropine which contains the same tetrahedral carbon, while the close analogue tropane benzoate, with a carbonyl linker, has high affinity at 5-HT_3_ receptors (63 nM; Fozard, 1989). We also found that the potent 5-HT_3_ receptor antagonist, granisetron, binds with a micromolar affinity at muscarinic receptors, suggesting that while general conformations of these ligands enable them to share common binding sites at both receptors, the linkers are likely to confer the key structural elements that drive receptor selectivity.

To find further evidence for the importance of this linker region, we performed docking into a homologue of the 5-HT_3_ receptor that has been co-crystallised with the antagonist granisetron in its binding site (Kesters et al., 2013). The predicted binding pose for the high affinity antagonist SDZ-ICT 322 was similar to the orientations of granisetron and tropisetron ligands in 5HTBP and AChBP co-crystal structures (Fig. 6E), which was anticipated given the similarity in their structures (Fig. 1) and affinities (Hibbs et al., 2009, Kesters et al., 2013). However, in scopolamine the tri-substituted tetrahedral carbon between the scopine tricyclic moiety and the aromatic phenyl ring leads to a kink in the molecular structure, unlike the high-affinity 5-HT_3_ receptor which are planar. In scopolamine this linker also contains a hydroxyl group. The docking results lead us to speculate that the substituted tetrahedral carbon in scopolamine creates increased bulk and ligand flexibility, while the polar hydroxyl group is sterically restricted and occupies a cavity in the rear of the binding site. If these predictions are correct, the differences in the linker region orientate scopolamine away from residues in binding loops D and F (Fig. 6D), and the ligand no longer engages with residues that are essential for high affinity binding (Thompson et al., 2005, Thompson et al., 2006).

Scopolamine is generally regarded as a non-selective muscarinic receptor antagonist with an affinity ≤1 nM. At higher concentrations it also blocks nicotinic acetylcholine receptors (*IC*_50_ = 928 μM) and increases the expression of α7 nACh receptors (Schmeller et al., 1995, Falsafi et al., 2012). When using scopolamine for the prevention of motion sickness in humans, blood concentrations following transdermal and combined oral administration have been reported to peak at ∼0.37 ng ml^−1^ within an hour (Nachum et al., 2001). Elsewhere, higher plasma concentrations of 2.9 ng ml^−1^ are reported following intravenous administration (0.4 mg) to healthy volunteers (Putcha et al., 1989). Both of these values are significantly lower than the concentrations that affect 5-HT_3_ receptors and it is unlikely that these receptors would be inhibited. However, when scopolamine is used to induce cognitive dysfunction in rodents, intraperitoneal or sub-cutaneous injections of up to 2 mg kg^−1^ are used (Klinkenberg and Blokland, 2010). As a weight per volume this is the equivalent of ∼1 μM which is close to the *IC*_50_ at 5-HT_3_ receptors. For centrally administered scopolamine the focal concentrations at the site of administration can be as high as 140 μg μl^−1^ (460 μM), a concentration that is far in excess of its *IC*_50_ at 5-HT_3_ receptors and would cause complete inhibition (Klinkenberg and Blokland, 2010).

The amygdala and hippocampus are of critical importance in implicit and explicit memory, and this function is mediated via actions of both cholinergic and serotonergic pathways. As scopolamine blocks muscarinic receptors with high affinity it is used to induce cognitive dysfunction, but it is also known that 5-HT_3_ receptor antagonists alleviate these symptoms. Long-term potentiation (LTP, the neural mechanism through which memory is formed) in the amygdala and hippocampus is inhibited by 5-HT_3_ receptor agonists and promoted by antagonists (Staubli and Xu, 1995). These effects are probably mediated via actions on the GABA-ergic synaptic activity of interneurons, but may also result from activities at 5-HT_3_ receptors that are present outside of the hippocampus and would also be blocked by systemically administered 5-HT_3_ antagonists. If sufficiently high concentrations of scopolamine were centrally administered we might expect a similar block of 5-HT_3_ receptors which could complicate the interpretation of its physiological effects. Pre-administering 5-HT_3_ antagonists to alleviate cognitive dysfunction might further complicate these studies as their higher affinities and slower elimination from the body would prevent scopolamine binding at 5-HT_3_ receptors (Putcha et al., 1989). As mood disorders such as anxiety and depression are also mediated by both cholinergic and serotonergic pathways, the interpretation of scopolamine effects on these might be similarly affected (Bétry et al., 2011).

In summary, we provide the first reported evidence that the drug scopolamine inhibits the function of homomeric 5-HT_3_ receptors via a competitive mode of action, and suggest that the bond that links the kinked and more flexible structure of scopolamine is responsible for the lower affinity when compared with other typically flat and rigid 5-HT_3_ receptor antagonists. Because the concentration of centrally administered scopolamine can exceed the concentration that inhibits 5-HT_3_ receptors, it is likely that these receptors would be inhibited under this experimental paradigm, and could influence LTP. Given this finding we believe that the potential effects at 5-HT_3_ receptors should be considered before centrally administering high concentrations of this compound.

## Conflicts of interest

There are no conflicts of interest arising from this work.

## Authorship contributions

Participated in research design: AJT.

Conducted experiments: AJT.

Contributed reagents or analytical tools: –

Performed data analysis: AJT, ML.

Wrote or contributed to the writing of the manuscript: AJT, ML.

## Figures and Tables

**Table 1:** Parameters derived from concentration-response curves in the presence of increasing concentrations of scopolamine.

[Scopolamine] (μM)	pEC_50_	EC_50_ (μM)	nH	n
Control	5.65 ± 0.02	2.24	2.1	6
10	5.49 ± 0.04	3.23	2.2	4
30	5.15 ± 0.01	7.08	3.3	4
60	4.87 ± 0.03	13.5	3.4	4
100	4.84 ± 0.04	14.4	3.9	3
300	4.36 ± 0.03	43.6	2.5	5
